# Frostbite injuries related to recreational nitrous oxide use: incidence, management, and complications in a Swedish case series

**DOI:** 10.1016/j.jpra.2024.07.019

**Published:** 2024-08-05

**Authors:** Yihang Liu, Karl Svennersten, David Schwartz, Fredrik Huss, Alberto Falk-Delgado

**Affiliations:** a Department of Molecular Medicine and Surgery, Karolinska Institutet, Stockholm, Sweden; b Department of Acute and Trauma Surgery, Karolinska University Hospital, Stockholm, Sweden; c Burn Centre, Department of Plastic and Maxillofacial Surgery, Uppsala University Hospital, Uppsala, Sweden; d Department of Surgical Sciences, Plastic Surgery, Uppsala University; e Department of Reconstructive Plastic Surgery, Karolinska University Hospital, Stockholm, Sweden

**Keywords:** Frost bite injury, Nitrous oxide, Surgery

## Abstract

**Background:**

Nitrous oxide (N_2_O) use in recreational settings has been increasing in Sweden and Europe and consequently, the related injuries are also increasing. We aimed to investigate the incidence, management, and surgical outcomes of frostbite injury (FI) related to N_2_O use.

**Material and Method:**

All patients in a 22-month period from 2021 to 2022 presenting with FI related to N_2_O abuse from 2 plastic surgery clinics (1 national burn center) were identified. Data regarding patient comorbidity, mechanism of injury, initial management, treatment, and follow-up were investigated. Complications following surgery were categorized into minor (treated in outpatient setting) and major (requiring reoperation) complications.

**Results:**

In total, 9 patients were identified; among them, 5 patients provided consent and were included in the study. Direct contact with the gas canister was the most common injury mechanism (n = 4). All but 1 patient contracted full-thickness injuries, and these 4 patients later required surgery. The medial thigh was the most common area of injury. Outpatient clinic visits were common (mean 4.8 visits/patient). Surgical complications were common and all operated patients were diagnosed with minor complications and half of them with major complications (wound dehiscence and scar contracture).

**Conclusion:**

Frostbite injuries arising from recreational N_2_O use are complex and often require surgical intervention that may lead to complications. With the increasing incidence of N_2_O abuse in Sweden, further research is crucial to address this emerging public health concerns and optimize treatment strategies for these distinctive injuries.

## Background

Nitrous oxide (N_2_O, dinitrogen oxide), also known as laughing gas, is a colorless non-flammable gas (at room temperature) with a slight sweet scent and taste.^1^

N_2_O can be used as rocket propellant, in combustion engines, and as a food additive (E942) specifically as an aerosol spray propellant for products such as whipped cream.^2^ In the medical community, N_2_O is mostly known for its anesthetic and pain-reducing effects in surgery and dentistry.^3^ Upon inhaling the gas, a euphoric (and slight hallucinogenic) effect is elicited. This euphoric effect is highly sought after in recreational use. Recreational use of N_2_O can be dated back to at least the 18th century “laughing gas parties” in the British upper class and this has increased in the 19th century, with the widespread availability of the gas, e.g., to be used for culinary purposes.

In Sweden, there has been a surge in the use of N_2_O as a recreational substance in recent years.^4^ Among students in their second year of high school, approximately 17% had experimented with N_2_O. For reference, similar numbers were presented for cannabis abuse.^4^ N_2_O can be stored in highly pressurized containers in liquid form. When the gas is released from its canister, the gas and canister quickly becomes extremely cold (approximately −40° C) owing to the Joule–Thomson effect and thus have the potential to cause severe frostbite injury (FI).^5^^,^^6^ Injuries can be sustained due to direct contact with the canister, commonly on the medial thighs or due to spillage of N_2_O commonly on the facial area or hands. When inhaling N_2_O directly from the canister, the high pressure can cause mechanical barotrauma to the lung tissue, leading to pneumothorax.^7^^,^^8^ Releasing the gas from the canister into a balloon, or similar containers, allowing the gas to warm up before inhaling, is a common procedure in recreational use. Besides physical damages, long-term N_2_O-abuse is also associated with neurotoxicity related to vitamin B12 deficiency and N-methyl-D-aspartate (NMDA) antagonism to which neonatal brains are most susceptible.^9^ Neurologic sequela in patients with long-term N_2_O abuse is also common with myeloneuropathy and peripheral neuropathy being the most common forms.^10^

FI may manifest in regions associated with winter sports, such as skiing, as well as in urban environments during winter, e.g., when individuals succumb to sleep in snowdrifts, frequently owing to intoxication. Additionally, instances of FI are observed in military or adventurous contexts. Although the management and treatment of FI mostly parallels that of burn injuries, several differences can be noted. FI, in contrast to traditional burn injuries, usually take longer to demark and could therefore, initially, be harder to diagnose correctly for the untrained. The tissue exposed to freezing temperatures respond via alternating cycles of vasoconstriction and vasodilatation.^11^ The result of this is partial thawing and refreezing phenomena that causes tissue damage. Furthermore, direct cell damage ensues after contact with the freezing agents due to the formation of extracellular ice crystals that damage the cell membranes.^12^ Treatment of FI is usually carried out in burn care centers and include rapid, but gentle, rewarming of the affected tissues, followed by surgical excision of necroses and skin grafting.^13^

Several burn centers around Europe have reported an increasing incidence of FI in relation to N_2_O use^5^^,^^6^^,^^14^ and our centers share the same perception, which is why this case series aimed to investigate the incidence, management, and follow-up of these patients in Sweden.

## Methods

Data from 2 university level plastic surgery clinics in Sweden (Uppsala University Hospital, Uppsala (1 of the 2 national burn care centers) and Karolinska University Hospital, Stockholm) were collected. Patients presenting with FI injury due to N_2_O use from January 1, 2021, to October 31, 2022, were identified and included. Patients presenting with FI injury without association with N_2_O were excluded. In total, 9 patients were identified and met the inclusion criteria; however, only 5 patients gave written informed consent and could be included.

Data on patient comorbidity, mechanism of injury, initial management, treatment, and follow-up were gathered from electronic medical charts by trained medical personnel.

Minor complications were defined as any complication (infection, wound dehiscence, and pain) that could be treated in an outpatient setting. Major complications were defined as complications that required unplanned revision surgery. Time to surgery was defined as time from injury to surgery.

## Results

### Overview of patients

In total, 5 patients were included (3 women, 2 men) (Table 1). Mean age was 23.4 years (range 20-32 years). All patients were previously healthy, but most of them smoked regularly. Direct contact with the N_2_O canister was the most common mechanism of injury. One patient received FI from spillage of N_2_O. Two patients were intoxicated with alcohol during injury.

**Table 1:** Overview of the included patients.

Patient ID	Sex	Age (y)	Comorbidity	Smoking	Mechanism of injury (MoI)	Intoxication at injury
1	Female	21	Nil	Yes	Direct contact with canister	Unknown
2	Male	32	Nil	Yes	Direct contact with canister	N_2_O + alcohol
3	Female	20	Nil	Yes	Direct contact with canister	N_2_O
4	Female	23	Nil	No	Leakage of N_2_O from canister	Alcohol
5	Male	21	Nil	No	Direct contact with canister	None

N_2_O: nitrous oxide.

### Injury and treatment

The most common site of injury was the inner thigh (n = 3, Table 2). One patient received injuries on the hand and the other on the face. Four of the 5 patients required surgical treatment due to full-thickness injuries (involving the muscle fascia). Total body surface area burnt ranged from 1-3.5%. Time from injury to presentation varied in the group, with a mean of 17 days (range 9-24 days). All but 1 of the operated patients needed more than 1 surgical intervention. One patient required cleaning and debridement of necroses before a split thickness skin graft could be applied to the wound. One patient needed dressing changes under anesthesia before definitive surgical intervention could be carried out.

**Table 2:** Management of FI, complications, and follow-up.

Patient ID	Anatomical site of FI	TBSA (%)	Depth	Management	Time to surgery (d)	Total operations (number)	Major complications	Minor complications	Hospital stay incl readmission (d)	Outpatient visit (number)
1	Inner thigh, bilateral	1	Full thickness	Surgical	19	3	1	1	2	5
2	Inner thigh, bilateral, anterior thigh, left	3.5	Full thickness	Surgical	14	1	0	1	6	6
3	Inner thigh, bilateral	2.5	Full thickness	Surgical	24	2	0	1	2	9
4	Hand, left	1	Full thickness	Surgical	11	2	1	1	1	2
5	Nose, eyebrow		Epidermal	Conservative	0	0	0	0	0	2

FI, frostbite injury.

All operated patients required hospital stay of at least 1 night (range 1-6 days, including readmissions) at the hospital. The whole group frequently visited the outpatient clinic, pre- and post-operatively (mean 4.8 visits/patient).

### Complications

Four out of 5 patients had complications and among the 4, 2 had major complications necessitating reoperations (1 wound dehiscence and 1 scar contracture).

### Follow-up

When followed-up 6 months after injury, 3 of the 4 patients who underwent operations had subjectively aesthetical problems with their scars. One patient had major scar contractures of the left digits III-V, which could not be treated sufficiently using conservative therapy and went through scar release with full-thickness skin transplant 21 months post-trauma.

## Discussion

This case series aimed to investigate the incidence, management, and results of FI related to recreational N_2_O use at 2 plastic surgery clinics in Sweden during a 22-month period.

Most of the included patients were young adults without comorbidities, who experimented with N_2_O, similar to the case series by Chen et al. on the same topic.^5^ making our studies comparable. For patients abusing N_2_O, the most common injury site was the medial thigh in ours and previous studies.^5^^,^^6^ This is probably due to patients holding the canister between their legs when releasing the gas. Reports have shown that larger, industrialized sized, canisters are often used in recreational settings, and therefore cause more damage.^15^ A majority of the cohort contracted deep tissue damage because of direct contact with the N_2_O canister and subsequently needed surgical intervention. Possible explanations for the deeper tissue damage could be the analgesic effect of the cold canister itself and analgesic effect from inhaling N_2_O that masks the pain at time of injury, likely prolonging the exposure time.

Surgery was needed in all but 1 case. As the time to surgery varied, besides patient delay, one could suspect “surgical timing” was difficult to determine. Three out of the 4 operated patients required more than 1 surgical intervention, which also highlights the complexity of the injury. As this type of injury is uncommon, diagnosis and treatment might not be as efficient as needed. This differed from the results of Chen et al., as in their cohort, 4 out of 16 were treated with surgical management.^5^ In the report by Hever et al., all patients were recommended surgery, but only 3 patients consented to surgery.^6^

**Figure 1:**
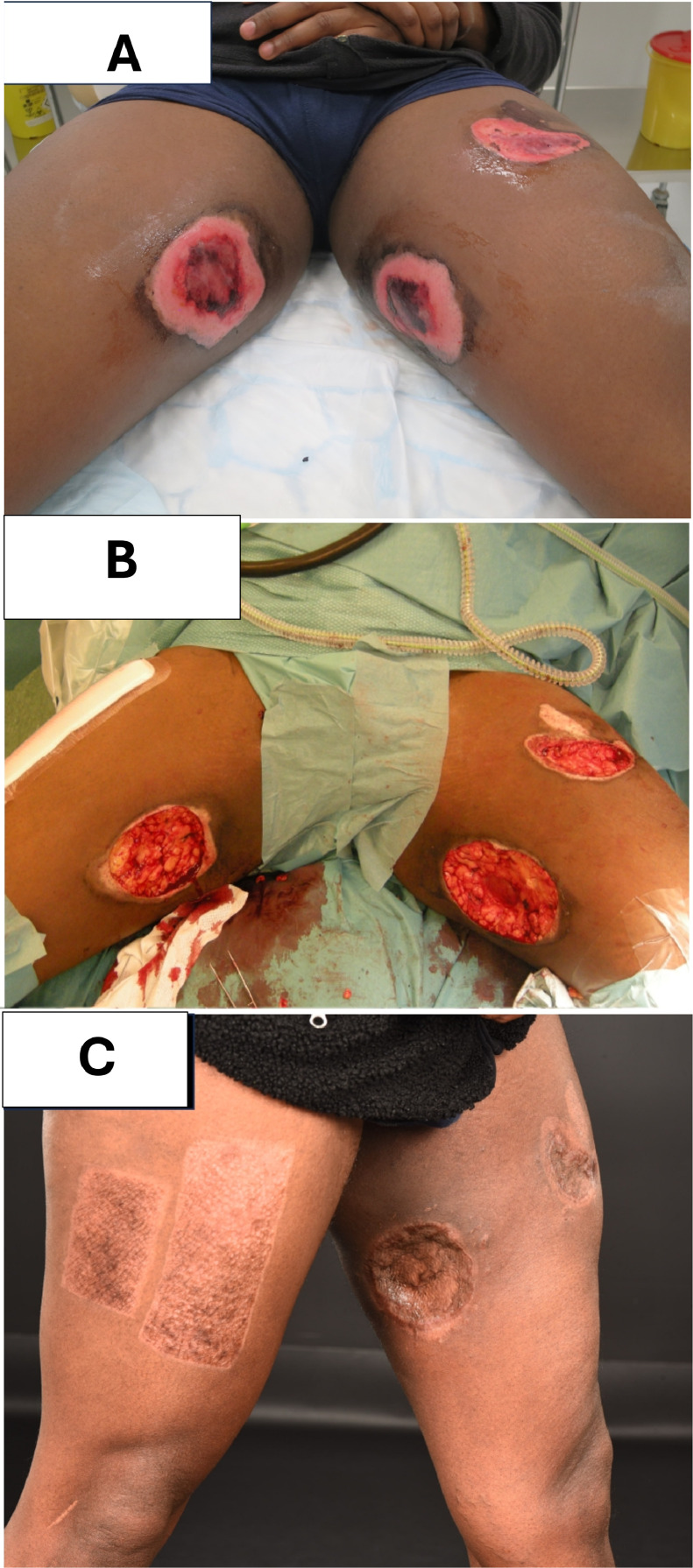
Clinical photographs of a 32-year-old male presenting with A) bilateral frostbite injuries to the inner thighs and ventral left thigh after contact with a large N_2_O cannister. B) Intraoperative photograph of revised injuries. C) 6-month follow-up.

**Figure 2:**
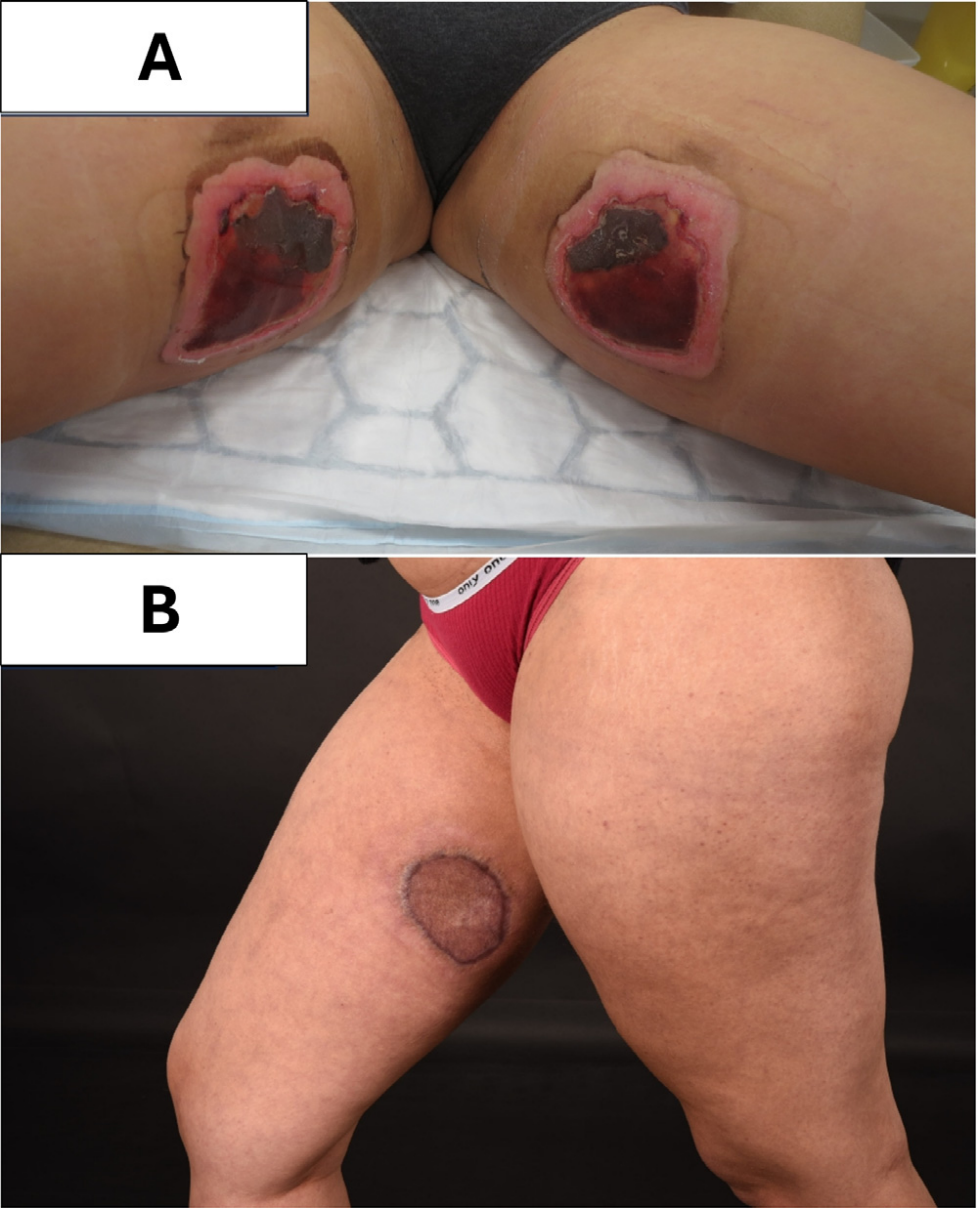
A) Clinical photograph of a 19 year old female presenting with bilateral frost bite injuries to the inner tighs after contact with a N_2_O cannister. B) 6-month follow-up after surgical debridement and split thickness skin grafting.

**Figure 3:**
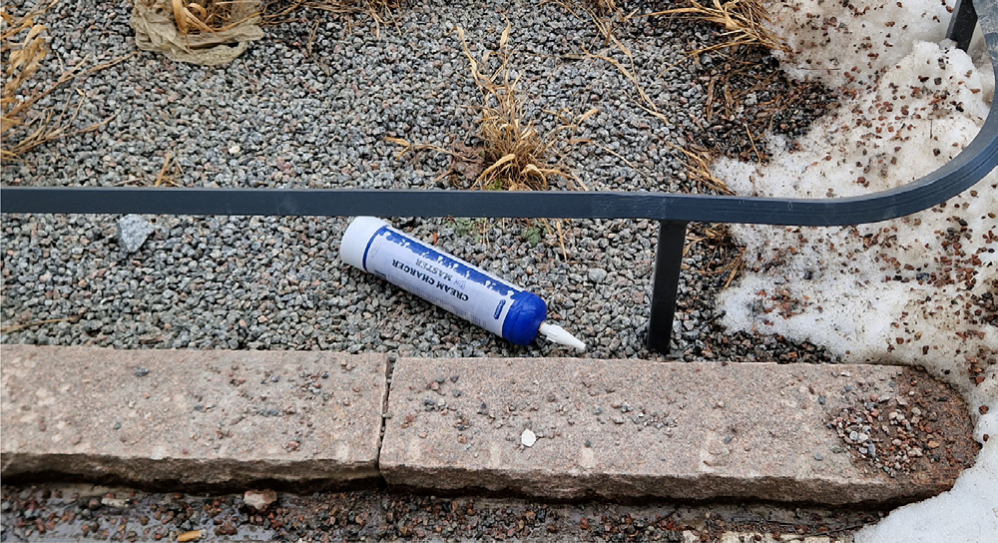
Larger sized N_2_O cannister.

We could not to assess the national incidence of such injuries as we did not have access to national data. However, N_2_O abuse has been shown to be on the rise in Sweden and therefore this injury mechanism is likely to be more common in the coming years.^16^^,^^17^ Our purpose with this case series is to illuminate the dangers of N_2_O abuse and complications leading to physical and cosmetic impairment. FI in N_2_O abusers can cause severe injuries that require multiple surgical and outpatient interventions. This highlights the need for more studies on the optimal surgical timing and efficiently treatment strategies for such injuries. We believe that preventative measures need to be taken, and all patients with tendencies to abuse should be recommended to contact a dependency disorder specialist (Figure 1, Figure 2, Figure 3).

## Conclusion

FI in patients using N_2_O can lead to deep and complex injuries that require surgical intervention and multiple outpatient visits. Complications arising from the surgery was a common motivating factor deserving further studies on the topic.

## Limitations of the Study

In this case series the limited number of patients is an evident weakness. Moreover, we could only include patients treated at the Karolinska and Uppsala University Hospitals and therefore selection bias is a possibility. Among the approximately 10 million inhabitants of Sweden, Karolinska treats patients from the Stockholm region (approximately 2 million inhabitants), and Uppsala treats patients mainly from the Uppsala region (approximately 500,000 inhabitants) but also serves as 1 of the 2 national burn care centers and therefore handles more severe cases.

## Conflict of Interests

None.
